# Combined Analysis of Multi-Study miRNA and mRNA Expression Data Shows Overlap of Selected miRNAs Involved in West Nile Virus Infections

**DOI:** 10.3390/genes15081030

**Published:** 2024-08-05

**Authors:** Franz Leonard Böge, Sergej Ruff, Shamini Hemandhar Kumar, Michael Selle, Stefanie Becker, Klaus Jung

**Affiliations:** 1 Institute of Animal Genomics, University of Veterinary Medicine Hannover, Bünteweg 17p, 30559 Hannover, Germany; franz.leonard.boege@tiho-hannover.de (F.L.B.); sergej.ruff@tiho-hannover.de (S.R.); shamini.hemandhar.kumar@tiho-hannover.de (S.H.K.); michael.selle@tiho-hannover.de (M.S.); 2 Institute of Parasitology, University of Veterinary Medicine Hannover, Bünteweg 17, 30539 Hannover, Germany; stefanie.becker@tiho-hannover.de

**Keywords:** gene-set test, microRNA, multi-omics, reproducibility, transcriptomics, West Nile Virus

## Abstract

The emerging zoonotic *West Nile virus* (WNV) has serious impact on public health. Thus, understanding the molecular basis of WNV infections in mammalian hosts is important to develop improved diagnostic and treatment strategies. In this context, the role of microRNAs (miRNAs) has been analyzed by several studies under different conditions and with different outcomes. A systematic comparison is therefore necessary. Furthermore, additional information from mRNA target expression data has rarely been taken into account to understand miRNA expression profiles under WNV infections. We conducted a meta-analysis of publicly available miRNA expression data from multiple independent studies, and analyzed them in a harmonized way to increase comparability. In addition, we used gene-set tests on mRNA target expression data to further gain evidence about differentially expressed miRNAs. For this purpose, we also studied the use of target information from different databases. We detected a substantial number of miRNA that emerged as differentially expressed from several miRNA datasets, and from the mRNA target data analysis as well. When using mRNA target data, we found that the targetscan databases provided the most useful information. We demonstrated improved miRNA detection through research synthesis of multiple independent miRNA datasets coupled with mRNA target set testing, leading to the discovery of multiple miRNAs which should be taken into account for further research on the molecular mechanism of WNV infections.

## 1. Introduction

The mosquito-transmitted *West Nile virus* (WNV; *Orthoflavius*, *Flaviviridae*) has significantly expanded its range in Europe within the past decade. Since its description in 1937 in Uganda, WNV has spread globally and is reported up to now from all continents except Antarctica. Autochthonous transmission of WNV has been established across Europe and also Germany since 2018 [1] leading to reoccurring outbreaks across Europe [2,3]. During the 2023 transmission season, 709 cases including 67 deaths were reported in Europe which is the third highest number per season after 2018 (1549 cases) and 2022 (1116 cases) [4]. These high case numbers highlight the importance of WNV as an emerging zoonotic disease. The virus perpetuates within an enzootic cycle involving ornithophilic mosquitoes (mostly of the *Culex pipiens* complex) as vectors and avian amplification hosts. Humans, equines, and other vertebrates can be infected [5,6] but are dead-end hosts due to low viremia [7]. WNV infections are usually asymptomatic but around 20% of patients develop West Nile fever (WNF) with clinical signs including headache, and mild febrile illness. Immunocompromised patients might develop West Nile neurological disease (WNND), including meningitis, encephalitis, or even death [8,9,10].

In the following, we exhibit the meaning of studying WNV on multiple omics levels, and give a short introduction on microRNAs and on our approach of using gene-set tests on the mRNA level to study miRNA alterations.

### 1.1. Studying West Nile Virus Infections on Multiple Omics Levels

WNV infections have been thoroughly studied on the mRNA transcriptome level [11]. However, it would be beneficial to gain further insight into the disease through information from other omics levels. Multi-omics analyses enable a deep understanding of the intricate interactions in biology including infections [12,13]. By integrating data from genomics, transcriptomics, proteomics, and other omics fields, underlying cellular processes can be discovered [14]. However, in molecular high-throughput studies, often only one omics level is studied. Still, based on the molecular interactions of the different omics levels between one another, changes of one omics level can be deducted from the data of another level. For instance, deciphering mRNA alterations can provide insights into potential proteomic shifts, enabling the anticipation of downstream effects [15].

### 1.2. MicroRNAs and Their Effect on Target mRNAs

In the context of infections such as WNV, the omics domain of microRNAs (miRNAs) is of particular interest. MiRNAs represent a class of endogenous, predominantly genomic encoded non-coding small RNAs with a typical length ranging from 18 to 24 nucleotides [16,17]. The miRNA biogenesis unfolds in both the nucleus and cytoplasm. After initial transcription of the approximately 70 base pair long pri-miRNA mediated by RNA polymerase II, the primary transcript [18,19] subsequently undergoes processing orchestrated by the Drosha and DGCR8 complex. The newly formed precursor miRNA (pre-miRNA) is then exported to the cytoplasm [18]. In the cytoplasm, pre-miRNAs undergo further refinement. Processed by the Dicer and RNase II, pre-miRNAs are transformed into double-stranded mature miRNAs, typically spanning around 22 nucleotides. The resultant mature miRNA functional strand then integrates into the RNA-induced silencing complex (RISC) cytoplasm [18]. Mature miRNAs then possess the ability of large-scale modulation of gene expression in the cell. They function through partial complementarity (mismatch) to their target mRNAs. Key to this interaction is the ‘seed sequence’, a critical element for the binding of miRNA to mRNA, strategically positioned 2–8 base pairs from the 5′-end of the miRNA. This sequence exhibits complementary pairing with the 3′-untranslated region (UTR) of the target mRNA, forming the molecular foundation for regulatory precision [20]. Through the only partial complementarity, required miRNAs can have a broad spectrum of mRNA targets. The down-regulation of targeted genes is relatively modest (∼30–50%). This enables single miRNA types to have a strong large-scale modulation effect on the cell’s proteome. This differentiates them from the function of siRNAs which have high complementarity to their single to a few targets which they strongly down-regulate (>90%). A substantial portion of the human transcriptome is targeted by at least one miRNA; so far, it is known for over 60% [21].

### 1.3. Using Gene-Set Tests on Target mRNAs to Study miRNA Expression Changes

Recognizing the limitations of traditional miRNA analysis, we employ a novel approach that is to infer changes on the miRNA level through mRNA data via gene-set testing such as gene-set enrichment analysis (GSEA). Hereby, sets of mRNA targets function as the gene-set in the gene-set testing. This not only provides a more comprehensive understanding but also surpasses the constraints of limited miRNA data. As we compare the outcomes of this innovative method with conventional differential analysis, our aim is to evaluate the efficacy and reliability of these approaches in the context of WNV infection.

Gene-set testing was originally used to ease the interpretation of large lists of differentially expressed genes. However, it can also be used to investigate correlations between different omics levels [22,23]. This approach involves grouping genes based on common functional annotations, pathways, or regulatory elements, shedding light on coordinated biological processes. In our study, gene-sets are formed by miRNA target genes. This not only aids in identifying potential regulatory relationships but also provides a nuanced understanding of how changes in miRNA levels may orchestrate mRNA expression patterns. In the past twenty years, a large number of approaches for gene-sets have been published, which can roughly be divided into three different types. ‘Self-contained’ tests rely on the expression matrix of the features in the set [24]. ‘Competitive’ tests (also termed ‘overrepresentation’ analysis) compare the proportions of set-assigned features in the differentially expressed genes and in the non-differentially ex-pressed genes. Finally, gene-set enrichment analysis studies the distribution of set-related features across all genes ranked by their *p*-value from differential expression analysis [25]. In our investigation, we make use of one representative test from each of these three categories.

The gene-set testing approach based on sets of mRNA target genes is first investigated in five datasets which include in parallel both high-throughput mRNA and miRNA expression profiles in WNV-infected samples and healthy controls. The investigation includes target set information from different public databases, different cutoff values that reflect the certainty of mRNAs to be true targets of a particular miRNA, as well as different methods for GSEA. Finally, we apply the most promising procedures to eight studies which only involve mRNA expression profiles.

## 2. Materials and Methods

In this section, we first present the mRNA and miRNA expression datasets selected from the ArrayExpress database (https://www.ebi.ac.uk/biostudies/arrayexpress, accessed on 1 December 2023) [26]. Next, we specify the methods for differential expression analysis on the level of mRNA and miRNA expression data, respectively. Finally, the approaches for target set testing are detailed. All analyses were conducted in the R programming environment (version 4.4.0, www.r-project.org).

### 2.1. Selected miRNA and mRNA Datasets

Data were sourced from ArrayExpress, a database for high-throughput functional genomics data. We searched for all studies related to mouse and WNV (search term: {“West Nile” AND mouse}), yielding 20 search results on December 2023. Of these, three were removed due to insufficient data or flawed experimental setup. Ten studies were part of sub-series within a super-series. We then identified studies containing both miRNA and mRNA transcriptomic data. Among the 13 studies, five, all part of the super-series, contained both miRNA and mRNA transcriptomic data (Table 1). The selection process is depicted in a diagram in the Supplementary Material following the PRISMA statement for meta-analyses. Notably, both types of data were not available on ArrayExpress for all five studies, but were available on NCBI GEO database (https://www.ncbi.nlm.nih.gov/geo/) [27]. So we used NCBI GEO database for additional acquisition. In the latter part of the project, an additional eight studies were selected which studied West Nile Virus-infected mice on an mRNA transcriptomic level (Table 2). Microarray platforms used to collect the mRNA and miRNA expression levels are specified in Supplementary Tables S1 and S2.

### 2.2. Differential Expression Analysis

To identify significant changes in miRNA expression associated with WNV infection in mice, a comprehensive differential expression analysis was conducted. The processed and normalized expression data obtained from the selected studies were subjected to differential expression analysis using the R-package ‘limma’ [34]. Normalization was carried out using the quantile method also implemented in the limma-package. Differential expression analysis was performed to compare the expression levels of individual miRNAs between WNV-infected mice and their respective control groups. miRNAs exhibiting statistically significant differential expression were identified based on False Discovery Rate (FDR)-adjusted *p*-values [35] and log2-fold change thresholds. This analysis not only facilitates the identification of key miRNAs implicated in the host response to WNV infection but also provides insights into the molecular mechanisms underlying the pathogenesis of the virus. Differential expression analysis as a prior step to gene-set analysis was run the same way on the mRNA expression data. In addition, we performed gene-set enrichment analysis among the differentially expressed mRNAs using the R-package ‘clusterProfiler’ [36] to study enrichment of KEGG pathways and GO terms.

### 2.3. Target Set Enrichment Analysis

To further investigate the role of miRNAs in WNV infections across omics levels, we also made use of mRNA expression profiles that were analyzed again by differential expression analysis and subsequent gene-set enrichment analysis. Hereby, the target genes of individual miRNAs were defined as the gene-sets. Pairs of miRNA × mRNA target gene-sets were taken from seven different target databases. We used the R-package ‘multimir’ [37] to download the target information from the following seven databases: miranda [38], mirtarbase [39], tarbase [40], pita [41], targetscan [42], mirdb [43], mirecords [44].

Among these, tarbase, mirtarbase, mirecords are databases relying on experimental evidence while the others are based on computational prediction algorithms. Those databases, relying on computationally predicted target sets, also provide prediction cutoffs (given in percent), which we also take into account in our analysis.

As mentioned in the introduction, we investigate representative different gene-set tests of three different categories. All three tests are well established gene-set tests that have been widely used in the analysis of high-throughput gene expression data. In detail, we employed the ROAST test [45] as a self-contained test, Fisher’s exact test [46] as a competitive gene-set test, and ROMER [47] as a GSEA-type test. While ROAST and ROMER are also implemented in the limma-package, we use the standard R-function fisher.test for the competitive gene-set analysis.

## 3. Results

In this section, we present the outcome of our investigation at different stages. First, we report the differentially expressed miRNAs directly selected from the five miRNA expression datasets. Next, we present the evaluation and performance of using target set testing with respect to different parameters such as target set databases, prediction cut-off or types of target set test. Then, we show the results from indirectly selecting miRNAs by target set testing over all 13 mRNA datasets. Finally, we detail the findings from differential analysis on the level of mRNA expression.

### 3.1. Differentially Expressed miRNAs Selected from miRNA Expression Data

**Figure 1:**
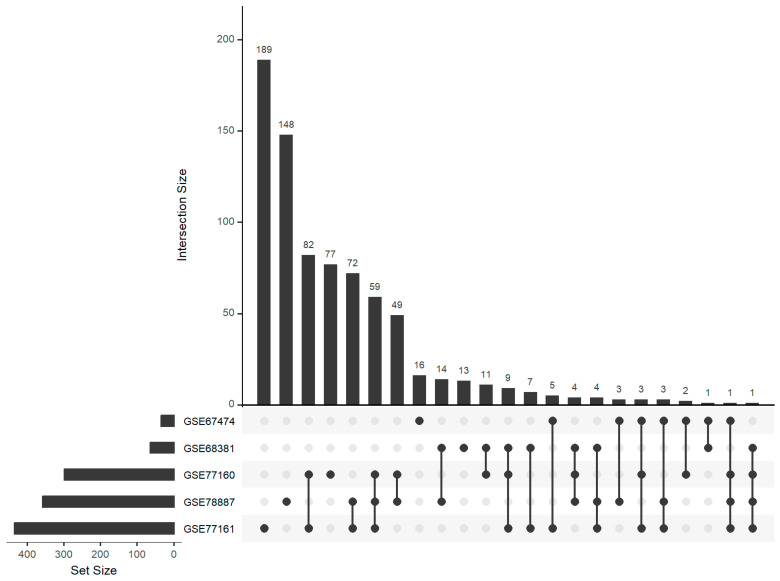
Numbers and overlaps between differentially expressed miRNAs selected from five independent miRNA expression datasets. The matrix is a grid where each row represents a unique combination of sets, and columns represent individual sets. Each bar of the lower bar graph corresponds to a set, and its height represents the size of that set; sets were ordered by size. The bars of the upper bar graph correspond to subset/intersections. The largest overlap was for miR-21a-3p and miR-297c-5p, which were differentially expressed in four datasets.

Through differential expression analysis across the five independent miRNA transcriptome datasets, we identified a number of differentially expressed miRNAs. In particular, the individual datasets resulted in 34 to 435 differentially expressed miRNAs (Table 3). Remarkably, our investigation unveiled a moderate degree of overlap among these datasets (Figure 1), wherein 84 differentially expressed miRNAs were found to be shared across at least three of the datasets. The largest overlap comprised 59 miRNAs which were shared in datasets GSE77160, GSE77161, and GSE78887. Noteworthy among these findings is that miR-21a-3p and miR-297c-5p were detected in four out of the five datasets, suggesting their potential significance in the infection of WNV. Furthermore, not only miR-21a-3p frequently occurred but also miR-21a-5p occurred in three of five datasets.

### 3.2. Running Tests for miRNA Using Target Enrichment Analysis and Differential Expression Analysis

To investigate the effects of WNV infection on the miRNA transcriptome, the target enrichment analysis was performed as stated in the methods section. This analysis was run using WNV parallel mRNA and miRNA expression datasets of WNV-infected mice, and based on the miRNA differential analysis from the previous subsection. Gene-set testing using mRNA target sets was performed on the five mRNA datasets related to the five miRNA datasets. Fisher’s exact test, ROAST, and ROMER procedures were used as target tests for the target set enrichment analysis. The *p*-values obtained from the miRNA differential analysis were subsequently correlated with the corresponding *p*-values from the mRNA target set tests using Pearson’s correlation coefficient R. More specifically, this correlation analysis was performed across a spectrum of 1400 conditions (280 conditions per dataset) encompassing the three different target set tests, different prediction cut-offs, and seven target set databases. Specifically, *p*-values were generated for each combination of target test (including Fisher’s exact, ROAST, ROMER), prediction cutoff (ranging from 5% to 99%), and target set database (mirdb, targetscan, mirecords, tarbase, mirtarbase, pita, and miranda). Most importantly, the analysis was performed for each condition once with the true miRNA databases’ target sets and once with randomly generated target sets that kept the same set size as a control. This enabled us to investigate if under any conditions the effect of using true target sets would significantly outperform random target sets.

**Figure 2:**
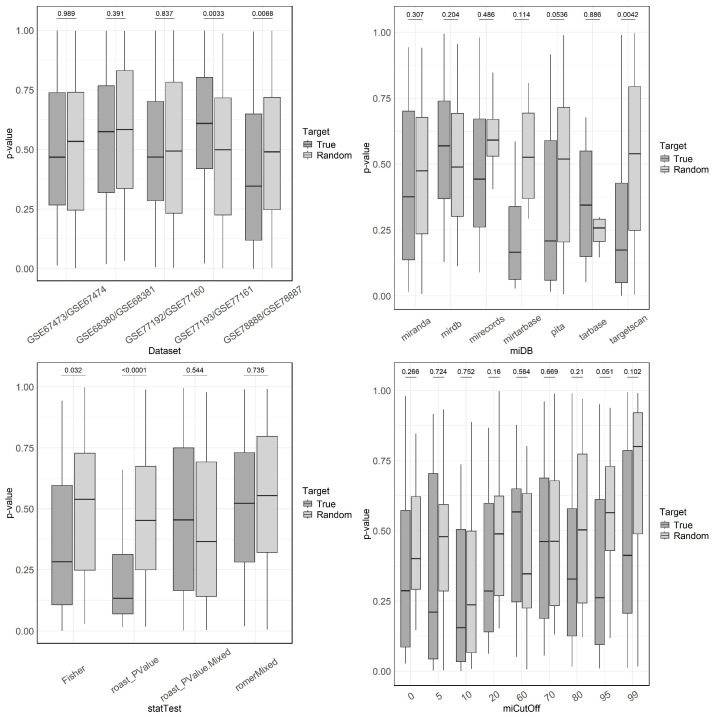
Correlation between mRNA- and miRNA-based (target set testing) predictions for differentially expressed miRNAs, comparing true target sets against random target sets. The top-left plot examines the effect of various datasets, showing variability in *p*-values, with GSE78888/GSE78887 demonstrating a notable difference between true and random sets. The top-right plot evaluates different miRNA target databases for the GSE78888/GSE78887 dataset, highlighting “targetscan” as particularly effective. The bottom-left plot compares statistical tests, with “roast_PValue” showing significant differences. The bottom-right plot assesses the impact of miRNA target set prediction cutoffs, showing a nearly significant difference between random and true target sets for a cutoff of 95% in the particular dataset.

First, we determined the correlation between miRNA testing and target set testing over all scenarios (databases, target set methods, and prediction cutoffs). Only the miRNA/mRNA-pair (GSE78887, GSE78888) showed overall significantly higher correlations when using true target sets compared to randomly selected target sets (Figure 2, top left). In the other four pairs of datasets, the correlation between miRNA and target set testing was overall weak and not stronger than with random target sets. Therefore, we found the results from these four pairs as not trustworthy, and used only the results from the pair (GSE78887, GSE78888) to select the most useful database, target test method, and prediction cutoff. Among the seven target databases, targetscan showed the largest difference between true and random target sets (Figure 2, top right). Among the target set methods, the method based on Fisher’s exact test and ROAST yielded the best results (Figure 2, bottom left). Finally, the analysis of prediction cutoffs showed no distinct trend, but with results peaking at a cutoff of 95% (Figure 2, bottom right). Therefore, we selected targetscan, Fisher, and a 95% cutoff as parameters for the selection of miRNA from the mRNA expression profiles as detailed in the next subsection. We opted to use Fisher’s exact test over ROAST, because the strong result only appeared for testing against the null-hypothesis that there is no difference, disregarding the fold change; for the mixed ROAST test, we received not as favorable results. However, since the evaluation of the target set testing approach did not provide a clear picture which of the pipeline settings would yield the best correlation with testing on the miRNA level, we also included a robustness analysis (Section 3.5) using another target database, different statistical test, and different prediction cutoffs.

### 3.3. Differentially Expressed miRNAs Selected from mRNA Target Set Testing

**Figure 3:**
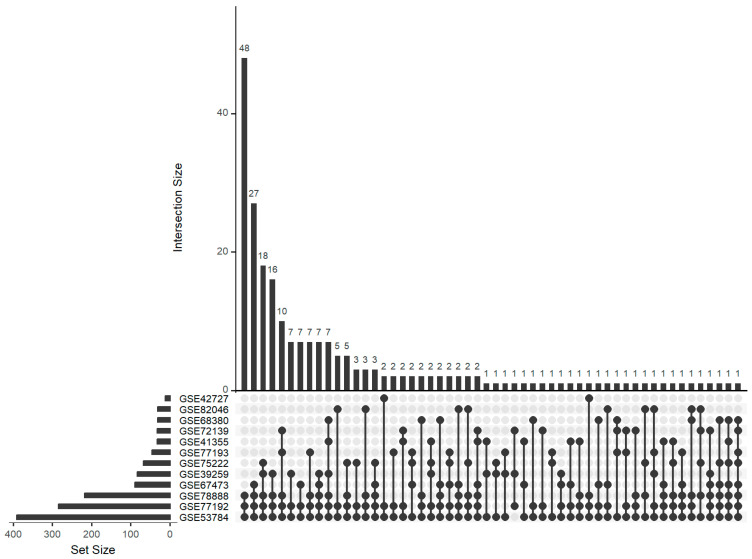
Comprehensive UpSet analysis of intersecting sets across thirteen WNV datasets. The matrix is a grid where each row represents a unique combination of sets, and columns represent individual sets. Each bar of the lower bar graph corresponds to a set, and its height represents the size of that set; sets were ordered by size. The bars of the upper bar graph correspond to subset/intersections. Only 12 out of 13 datasets shown as one did not contain any overlaps.

Following the realization that individual target set tests yielded insufficient information, a deeper analysis of overlapping findings from multiple independent studies became imperative. A comprehensive UpSet diagram analysis was conducted to assess the intersections across eight studies with only mRNA data available plus the five mRNA datasets where parallel miRNA data were available (Figure 3). Screening these 13 datasets, from mice infected with WNV, revealed recurrent emergence of several miRNAs. Notably, miR-325-3p was common among 8 out of the 13 datasets and miR-124-3p.1 was common among 7. Eleven further miRNAs were found in exactly six different datasets. Overall, there were 223 miRNAs that were selected in at least three datasets. Our analysis identified enriched miRNA target sets, providing valuable clues about the regulatory interactions between miRNAs and their target mRNAs during viral pathogenesis. Functional annotation of enriched miRNA target sets offered insights into the biological processes and pathways modulated by miRNAs in response to WNV infection.

### 3.4. Overlap of Findings from miRNA Analysis and Target Set Testing

Examining the overlap between miRNA analysis and target set testing, we focused on miRNAs identified in at least three datasets by both approaches to ensure robustness.

Taking the 84 miRNAs selected by miRNA testing in at least 3 of 5 datasets and the 223 miRNAs selected by target set testing in at least 3 of 13 datasets, our investigation revealed 13 miRNAs selected by both approaches. We conducted a search for publications on PubMed (Table 4) and Google Scholar (Supplementary Table S3) looking for articles where one of the 13 miRNAs was named in the title together with specified keywords related to WNV, inflammation or infectious diseases. Specifically, the PubMed analysis yielded 13 miRNAs associated with inflammation and 10 with viral activity, while Google Scholar yielded 11 miRNA related to inflammation and 7 related to viral activity. In addition, publications on five of these miRNAs contained the term neuroinflammation and three contained the term encephalitis in the PubMed analysis. These findings underscore the potential involvement of these miRNAs in West Nile virus infection, although we did not detect articles that refer to these miRNAs in the context of WNV.

### 3.5. Robustness Analysis of miRNAs Selected by Target Set Testing

Since the evaluation of the target set testing approach did not yield a clear winner regarding target database, statistical test, and prediction cutoff, we reran our pipeline to see which of the 13 miRNAs from Table 4 would have been also selected when choosing a different setting. Specifically, we reran the analysis additionally with mirdb as the target database, ROMER as the target set test, and a prediction cutoff of 80%. In total, we generated target set testing results with eight different settings. Two miRNAs, miR-15b-5p and miR-363-3p, were identified consistently in all eight settings, while miR-21a-5p was only selected in the primary setting (Supplementary Table S4). Besides these extremes, the remaining 10 miRNAs were selected in two to seven settings.

### 3.6. Differential Expression Analysis on the mRNA Level

In order to investigate what effects the expression changes on the miRNA level might cause on the mRNA level, we analyzed the expression changes on the mRNA level in more detail. The amount of differentially expressed genes according to different selection criteria was very different among the thirteen datasets (Table 5). For the parallel miRNA-mRNA datasets, we found that large effects on the miRNA level do not necessarily cause large effects on the mRNA level. For example, a large number of differentially expressed miRNAs in dataset GSE78887 (53 of 1247 miRNAs) corresponded to an only moderate expression change on the mRNA level in dataset GSE78888 (719 of 24,162 mRNAs). Vice versa, 4172 differentially expressed mRNA were observed in dataset GSE77192, but only 5 differentially expressed miRNAs were observed in the parallel dataset.

Focusing on individual genes, there were two mRNAs that were selected as differentially expressed in ten of thirteen datasets: IFIT3 (Interferon-induced protein with tetratricopeptide repeats 3) and RTP4 (Receptor Transporter Protein 4). Additionally, twelve mRNAs were selected in nine of the datasets: *CXCL10*, *DDX58*, *GBP2*, *IFITM3*, *IGTP*, *LY6A*, *MX2*, *OAS2*, *OASL1*, *OASL2*, *PHF11*, *USP18*.

For the above-mentioned 14 mRNAs, we checked whether these are target genes of the 13 miRNAs listed in Table 4, but only *CXCL10* was listed in targetscan as a target of miR-21a-5p and *MX2* as a target of miR-17-5p. Considering the stringent selection process for mRNAs and miRNAs, these two connections are the strongest associations we could identify.

The KEGG pathway and GO term enrichment analysis were based on the mRNAs selected by FDR-adjusted *p*-values < 0.05 and an absolute log2 fold change > 1 (Table 5). The results of enrichment analysis from the 13 datasets were aggregated using the R-package ‘RobustRankAggreg’ [48] yielding a score by which pathways and GO terms can be ranked. Among the top 20 KEGG pathways, we found many related to other infectious diseases such as Influenza A, Epstein–Barr virus infection and Herpes simplex virus 1 infection; furthermore, the Toll-like receptor signaling pathway and the Cytosolic DNA-sensing pathway both play a major role in the innate immune system. Among the top 20 GO terms, we found 2 terms related to cytokine activity (GO:0005125) and cytokine receptor binding (GO:0005126) as well as type I interferon receptor binding (GO:0005132), all of which are also related to immune response. Full enrichment results are provided in Supplementary Tables S3 and S4.

## 4. Discussion

As several studies, particularly those from which the data were used in our meta-analysis, have demonstrated, miRNAs are affected by WNV infection [49,50,51] and should be considered when deciphering the molecular mechanisms underlying WNV-induced disease. Findings of these previous studies have been inconsistent, often due to variations in experimental designs and methods. Recognizing this, our aim was to reanalyze the existing data in a harmonized way to identify consistent overlaps that could be deemed as a robust finding.

By combining insights from multiple studies as well as multiple omics levels, we aimed to increase the reproducibility and robustness when identifying miRNAs that are associated with WNV infection. While we identified miRNAs that are differentially expressed through the conventional way of differential expression analysis of the miRNA expression data, we also performed target set testing on mRNA expression data, grouping mRNA targets based on their miRNA target sets. By seeking intersections between the miRNAs identified through differential expression analysis and those identified through target set analysis across multiple datasets, we aim to robustly predict miRNAs involved in WNV infection. This allows us to gain deeper insights into the molecular mechanisms underlying WNV infection and offers a more reliable understanding of the role of miRNAs in this disease.

The differential expression analysis showed across the five miRNA transcriptome datasets a varying degree of differentially expressed miRNAs from GSE78887, GSE161, and GSE77160 yielding 359, 435, and 298, respectively; GSE68281 and GSE67474 had lower yields with only 64 and 34, respectively, which reduced to 1 and 2 for adjusted *p*-values. Between the datasets, there was a moderate degree of overlap with 84 different miRNAs that could be found in at least three of the five datasets. Fifty-nine of these miRNAs would be common to GSE77160, GSE77161, and GSE78887. Of greatest interest, here, are miR-21a-3p and miR-297c-5p for each being found in four of the five datasets, and miR-21a-5p for not only being found in three out of the five datasets but also being the other miRNA of the miRNA duplex with miR-21a-3p.

In our study, we formulated a target set testing approach for miRNAs using mRNAs by modifying a gene-set enrichment test with miRNA target sets. Of course, it was a limitation to have only a series of parallel miRNA-mRNA datasets from the same authors available to evaluate the approach of target set testing. On the other hand, we gained more robustness by including an additional eight mRNA datasets from other authors. Through optimization of parameters—type of target set test, miRNA target database, and prediction cutoff—to we aimed to enhance the accuracy and reliability of our analysis. Our results revealed that the miRNA target set databases that are chosen have a significant effect on the outcome. Interestingly, provided a decent quality of the dataset, targetscan outperformed other databases, despite being primarily based on computational predictions rather than experimental validation. This finding underscores the importance in assessing the different online available resources for more success in analysis in the future. In addition, our investigation revealed that prediction cutoffs of around 95% yielded a positive effect, suggesting the potential benefit of employing lower cutoff thresholds in similar analyses. In our study, the best performing statistical tests were Fisher’s exact test and ROAST. While these findings were intriguing, testing a new computational approach and optimizing key parameters laid only the groundwork for more precise and effective miRNA target set testing methodologies. The different types of set-based analysis may also lead to different interpretations of the results. We refer to comparative analyses of different types of enrichment and set-based analysis [52,53].

Initial target set tests did not yield consistent enough information on miRNAs involved in WNV infection. In response, we opted for a more comprehensive approach by focusing on intersects from multiple independent studies. Using an UpSet plot, we examined intersects across 13 datasets. This screening of datasets with consecutive analysis of the overlaps revealed several recurrent miRNAs. Notably, mmu-miR-124-3p.1 emerged in 8 out of the 13 datasets, and mmu-miR-325-3 was present in 7 datasets. Additionally, we identified 10 miRNAs that appeared in exactly six datasets.

To predict miRNAs involved in WNV infection, we analyzed the intersection between miRNA analysis and target set testing, focusing on miRNAs identified in at least three datasets by both methods. We discovered 13 miRNAs that were consistently associated with both approaches. Literature research revealed significant connections between these miRNAs and inflammation or viral activity neuroinflammation, further emphasizing their potential role in WNV infection. Noteworthy were miR-17-5p, miR-21a-5p, and let-7a-5p, which emerged as the most notable candidates, with multiple studies linking them to viral infection, inflammation, and even neuroinflammation and encephalitis albeit not specifically to WNV infection as of yet [54]. To make a fair interpretation, we must add that the studies referred to in Table 4 present the miRNAs under very different background and with focus on different diseases. Therefore, the conclusions taken by our bibliometric analysis are limited in that way that we can just observe that the miRNAs were identified in other infection or inflammation contexts than WNV. A direct comparison of the direction of deregulation is however not possible.

Let-7a-5p has been shown to play a role in inflammatory and viral contexts. Let-7a-5p is positively regulating important immune-related genes such as *TLR3* (Toll-Like Receptor 3), *RIG-I* (Retinoic Acid-Inducible Gene I), and *MDA5* [55]. It has been found to be down-regulated in EBV [56] and it is likely to be a key regulator of inflammation through proinflammatory macrophage activation in the lung following ozone exposure [57]. These findings in combination with its association with neuroinflammation make it an interesting therapeutic target [48]. Furthermore, let-7a-5p was described in the context of Japanese encephalitis virus (JEV) infection [58], a virus closely related to WNV (belonging to the same serogroup). In JEV infection, an induction of let-7a led to neuronal death via caspase activation, linking this miRNA to neuroinflammation.

Similar to Let-7a-5p, miR-17-5p also seems to induce pathological effects. Studies link miR-17-5p to inflammation and to a multitude of viruses. Inhibition of miR-17-5p increases *TXNIP* expression promoting apoptosis. By binding to *NLRP3*, *TXNIP* initiates an inflammatory reaction [59]. Not only is miR-17-5p suggested to be involved in the damage caused by hepatitis C virus (HCV) and COVID-19, but it is also hypothesized that the absence of *HT22*, which accumulates miR-17-5p, acts as a neuroprotective regulator of inflammation [60,61,62]. miR-21 has also been described to be up-regulated in the context of Dengue virus infection [63] and to promote viral infection although the mechanisms behind this are not yet understood.

The miRNA duplex miR-21a-5p/miR-21a-3p is also associated with viruses and inflammation so miR-21a-5p has been shown to facilitate virus proliferation by down-regulating Caskin1, a direct target gene, while miR-21a-3p has been showcased for its protective effects under inflammatory conditions, promoting WNV infection [64,65].

Interestingly, by targeting *GLRX*, miR-132-3p exerts a toxic effect on neurons through increasing the activation of microglia cells as well as promoting the release of inflammatory cytokines. Through this neuroinflammatory effect, it has been associated with Parkinson’s disease and Alzheimer’s disease [66,67].

Upon LPS induction of macrophages, miR-212-3p is up-regulated. It functions as a negative regulator of inflammatory cytokine production. It inhibits the expression of pro-inflammatory cytokines, such as *TNF-α* and *IL-6*, by targeting the 3′-UTR region of HMGB1. Through this, miR-212-3p suppresses for the inflammatory response important to p38 MAPK and ERK signaling pathways [68].

Understanding the role of these miRNAs in inflammatory and viral contexts offers valuable insights into the WNV infection. Seeing how multiple of the named miRNAs show an association with macrophage activation in the context of neuroinflammation can highlight more how neuropathological symptoms arise, especially miR-132-3p, which is already associated with progressive neurological diseases such as Alzheimer’s, which shows a potential mechanism into how similar symptoms arise in WNV infection. Furthermore, by showcasing how miR-21a-5p is associated with virus proliferation in related viruses, this could be an interesting future endeavor into potential miRNA therapeutics for WNV.

Among the mRNAs most frequently identified in our meta-analysis, many have already been mentioned in the context of WNV or other Flaviviridae, especially *MX2* and **CXCL10**, which are targets of miR-17-5p and miR-21a-5p, respectively, which were detected in another RNA-seq of mouse samples [69] and in a transcriptomic meta-analysis [11]. Also, the interferons *IFITM3* and *IFIT3* were multiple times named to play a significant role in WNV infection [70,71]. Finally, the oligoadenylate synthetases (*OAS1* and *OAS2*) as well as the *OAS*-like proteins *OASL1* and *OASL2* were regularly found to be deregulated by WNV infection [72].

The relationship between miRNAs and their mRNA targets presents certain challenges, as highlighted by [47]. These findings are consistent with many other problems to find correlations between different omics levels. Despite these challenges, our study takes a conservative approach by trying to find intersections between multiple methods. This rigorous approach ensures that the complexity and difficulties do not diminish the strength of our claims about the therapeutic interest of these miRNAs. By requiring that an miRNA was selected in our meta-analysis in multiple studies and by different analysis techniques, we allowed rather for an increased risk of false negatives than of false positives.

Consistently identifying 13 miRNAs across multiple datasets is particularly significant, as numerous studies have linked the selected miRNAs already to viral infection, inflammation, and neuroinflammation. That no previous studies linked them to WNV is not discouraging as the disparity in research volume between different viral infections can be huge. As of April 2024, there are approximately 2,650,000 studies on COVID-19 compared to 416,000 studies on WNV available on “Google Scholar”. This significant difference in the number of studies likely contributes to the greater availability of data and findings related to COVID-19. So this should not detract from the relevance of our identified miRNAs, which are implicated in viral pathogenesis and inflammation in other contexts and could play similar roles in WNV infection.

Having used expression data from mouse studies, it may be that our biological findings are not transferable to other—more natural—hosts of West Nile virus. However, our selected miRNAs and related mRNA targets can function as an anchor point for further research on WNV infection in other host species.

Additionally, it is worth noting that the datasets used had heterogeneous tissue types and platforms to collect the data. While usually this might be viewed as a limitation in a different setup of a meta-analysis, it was less a problem in this setup as since our primary objective was to identify “robustly” miRNA associated with WNV infection independent of tissue type. By focusing on recurrent miRNAs across multiple datasets of various tissue types and integrating the results from both miRNA and mRNA analyses, we aimed to achieve more consistent and reliable findings.

Overall, we think that more meta-analyses or other forms of evidence synthesis are necessary in the omics fields. As was pointed out by Gurevitch et al. (2018) [73], “evidence synthesis should become a regular companion to primary scientific research to maximize the effectiveness of scientific inquiry”. In omics research, much more raw datasets are freely available compared to other scientific disciplines. Therefore, besides the combination of analysis results (as typical in classical meta-analysis), raw data can either be fused and jointly modeled or at least be analyzed in a harmonized way. However, the opportunity of research synthesis has been little used in omics research so far. Although several methods for meta-analysis of omics data have been presented, doing a concrete meta-analysis is usually not straightforward due to usually having data from different sources and under different conditions. Najagawa et al. [74] provide a critical meta-evaluation of meta-analysis in the biological context. If carefully conducted, using parallel data from other omics levels can therefore help to increase the evidence of findings.

## Figures and Tables

**Table 1:** Summary of selected public microarray datasets for mRNA expression analysis in response to West Nile Virus (WNV) infection.

Databank ID	Mouse Line	Tissue	Time after Infection	Sample Size (Control + WNV)	Reference	Omics Level
GSE39259	n.a.	Liver	4 days	2 + 3	[28]	mRNA
GSE41355	n.a.	Bone marrow-derived dendritic cells	24 h	3 + 3	[29]	mRNA
GSE42727	C57BL/6	Cortical and granule neuron	12 + 24 h	10 + 10	[30]	mRNA
GSE53784	SW	Whole brain	n.a.	3 + 3	[31]	mRNA
GSE72139	C57Bl/6	Hippocampus	25 days	4 + 8	[32]	mRNA
GSE74628	C57BL/6	Primary myeloid dendritic cell	24 h	2 + 3	[33]	mRNA
GSE75222	C57BL/6	Primary myeloid dendritic cell	24 h	3 + 2	[33]	mRNA
GSE82046	crossline genetic background	Spleen	28 days	19 + 16	n.a.	mRNA

**Table 2:** Summary of selected public microarray datasets from studies on West Nile Virus (WNV) infection containing parallel miRNA-mRNA expression profiles, miRNA dataset being shaded.

Databank ID	Mouse Line	Tissue	Time after Infection	Sample Size (Control + WNV)	Reference	Omics Level
GSE67473	C57Bl/6	Cortical neuron	24 h	6 + 6	[33]	mRNA
GSE67474	C57Bl/6	Cortical neuron	12 h	5 + 6	[33]	miRNA
GSE68380	C57Bl/6	Granule cell neurons	24 h	6 + 6	[33]	mRNA
GSE68381	C57Bl/6	Granule cell neurons	12 h	6 + 6	[33]	miRNA
GSE77192	C57Bl/6	Cerebellum	4 days	5 + 5	[33]	mRNA
GSE77160	C57Bl/6	Cerebellum	4 days	5 + 5	[33]	miRNA
GSE77193	C57BL/6	Cortex	4 days	5 + 5	[33]	mRNA
GSE77161	C57BL/6	Cortex	4 days	5 + 5	[33]	miRNA
GSE78888	C57BL/6	Popliteal lymph node	1 days	3 + 5	[33]	mRNA
GSE78887	C57BL/6	Popliteal lymph node	2 days	3 + 5	[33]	miRNA

**Table 3:** Summary of differentially expressed miRNAs across five independent miRNA transcriptome datasets following West Nile Virus (WNV) infection. The #-sign stands for ‘number of’.

Databank ID	# miRNA in Dataset	# *p* < 0.05	# pFDR < 0.05	# |log2FC| > 1
GSE78887	1247	359	199	53
GSE77161	1247	435	119	13
GSE77160	1247	298	59	5
GSE68381	1247	64	1	2
GSE67474	1247	34	2	0

**Table 4:** Occurrences of keywords in article titles based on a PubMed search for each of 13 miRNAs selected on the miRNA level and by target set testing. Each row corresponds to a specific miRNA, and the columns represent search terms used to query titles of publications. The numbers indicate how many times each search term appeared in the titles of publications associated with the respective miRNA.

	WNV	West Nile	Virus	Viral	HIV	Covid	Inflammation	Inflammatory	Encephalitis	Neuroinflammation	Hits
miR-17-5p	0	0	13	10	1	5	13	5	0	1	48
let-7a-5p	0	0	5	3	1	3	3	8	0	1	24
miR-15b-5p	0	0	2	2	1	3	3	7	0	0	18
miR-132-3p	0	0	3	0	0	0	6	5	1	2	17
miR-185-5p	0	0	4	0	0	2	3	6	0	0	15
miR-21a-5p	0	0	1	1	0	0	7	3	0	2	14
miR-381-3p	0	0	3	0	1	0	6	1	2	0	13
miR-92a-3p	0	0	1	1	0	5	1	1	1	0	10
miR-212-3p	0	0	0	0	0	1	3	4	0	1	9
miR-18a-5p	0	0	1	0	1	2	3	1	0	0	8
miR-363-3p	0	0	2	1	0	0	1	2	0	0	6
miR-665-3p	0	0	0	0	0	0	2	1	0	0	3
miR-7a-5p	0	0	0	0	0	0	3	0	0	0	3

**Table 5:** Summary of differentially expressed mRNAs across thirteen independent mRNA transcriptome datasets following West Nile Virus (WNV) infection. The #-sign stands for ‘number of’.

**Databank ID** **of mRNA Data**	**Databank ID of Parallel miRNA Data**	**# mRNA in** **Dataset**	**# *p* < 0.05**	**# pFDR < 0.05**	**# |log2FC| > 1**
GSE39259		21,200	827	0	0
GSE41355		18,120	3479	1510	211
GSE42727		21,609	544	0	0
GSE53784		24,214	9231	6409	781
GSE67473	GSE67474	24,162	4048	1335	210
GSE68380	GSE68381	24,159	1067	140	78
GSE72139		30,869	3786	534	346
GSE74628		24,162	6405	1552	719
GSE75222		24,162	3195	81	73
GSE77192	GSE77160	24,162	14,981	13,618	4172
GSE77193	GSE77161	24,162	9356	4971	661
GSE78888	GSE78887	24,162	6812	2969	719
GSE82046		23,736	1070	1	1

## Data Availability

All miRNA and mRNA expression datasets analyzed in this study and listed in Table 1 and Table 2 are publicly available from the Gene Expression Omnibus database (https://www.ncbi.nlm.nih.gov/geo/).

## Supplementary Materials

The following supporting information can be downloaded at: https://www.mdpi.com/article/10.3390/genes15081030/s1, Table S1: Microarray platforms used for taking the mRNA expression levels in the individual studies; Table S2: Microarray platforms used for taking the mRNA and miRNA expression levels in the individual studies; Table S3: Occurrences of keywords in article titles based on a Google Scholar search for each 13 miRNAs selected on the miRNA level and by target set testing. Each row corresponds to a specific miRNA, and the columns represent search terms used to query titles of publications. The numbers indicate how many times each search term appeared in the titles of publications associated with the respective miRNA; Table S4: Robustness analysis of miRNAs selected by target set testing. The main analysis by target set testing was performed using target set information from the TargetScan database, Fisher’s test and an prediction threshold of 95%, yielding 13 miRNAs that were also selected on the level of miRNA expression. The “X” symbol marks miRNAs that were also selected by other parameter settings of the target set testing pipeline; Table S5: KEGG enrichment results; Table S6: GO term enrichment results. Figure S1: PRISMA workflow for selection of studies to be included in the meta-analysis.
