# Supplementary material for: Combined Analysis of Multi-Study miRNA and mRNA Expression Data Shows Overlap of Selected miRNAs Involved in West Nile Virus Infections
Source: Genes (Basel). 2024 Aug 5;15(8):1030. doi: 10.3390/genes15081030 (PMC11353516; doi:10.3390/genes15081030)
Supplement: Supplementary file 1 [file genes-15-01030-s001.zip › Supplemental_Materials.pdf]

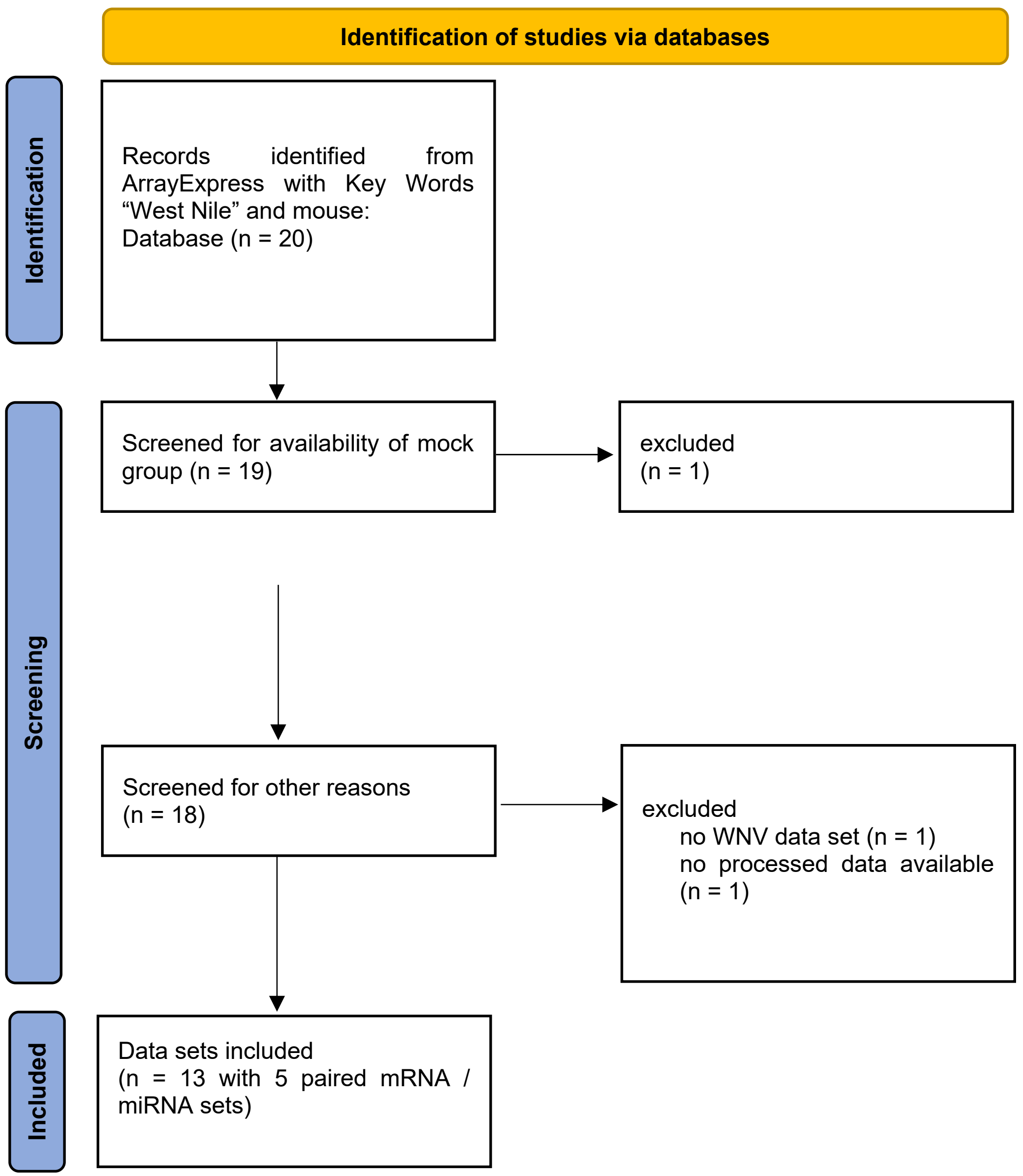

Supplementary Figure S1: PRISMA workflow for selection of studies to be included in the meta-analysis.

Databank ID	Platform
GSE39259	Agilent-014868 Whole Mouse Genome Microarray 4x44K G4122F (Probe Name version)
GSE41355	Illumina MouseRef-8 v2.0 expression beadchip
GSE42727	Agilent-014868 Whole Mouse Genome Microarray 4x44K G4122F (Feature Number version)
GSE53784	[MoGene-1_0-st] Affymetrix Mouse Gene 1.0 ST Array [transcript (gene) version]
GSE72139	Illumina MouseWG-6 v2.0 expression beadchip
GSE74628	Agilent-026655 Whole Mouse Genome Microarray 4x44K v2 (Probe Name version)
GSE75222	Agilent-026655 Whole Mouse Genome Microarray 4x44K v2 (Probe Name version)
GSE82046	[MoGene-2_1-st] Affymetrix Mouse Gene 2.1 ST Array [transcript (gene) version]

Supplementary Table S1: Microarray platforms used for taking the mRNA expression levels in the individual studies.

Databank ID	Platform
GSE67473	Agilent-026655 Whole Mouse Genome Microarray 4x44K v2 (Probe Name version)
GSE67474	Agilent-046065 Mouse_miRNA_V19.0_Microarray [Probe Name version]
GSE68380	Agilent-026655 Whole Mouse Genome Microarray 4x44K v2 (Probe Name version)
GSE68381	Agilent-046065 Mouse_miRNA_V19.0_Microarray [Probe Name version]
GSE77192	Agilent-026655 Whole Mouse Genome Microarray 4x44K v2 (Probe Name version)
GSE77160	Agilent-046065 Mouse_miRNA_V19.0_Microarray [Probe Name version]
GSE77193	Agilent-026655 Whole Mouse Genome Microarray 4x44K v2 (Probe Name version)
GSE77161	Agilent-046065 Mouse_miRNA_V19.0_Microarray [Probe Name version]
GSE78888	Agilent-026655 Whole Mouse Genome Microarray 4x44K v2 (Probe Name version)
GSE78887	Agilent-046065 Mouse_miRNA_V19.0_Microarray [Probe Name version]

Supplementary Table S2: Microarray platforms used for taking the mRNA and miRNA expression levels in the individual studies.

	WNV	West Nile	Virus	Viral	HIV	Covid	Inflammation	Inflammatory	Encephalitis	Neuroinflammation	Hits
miR-17-5p	0	0	1	0	0	1	13	2	0	0	**17**
let-7a-5p	0	0	0	1	0	0	2	2	0	0	**5**
miR-15b-5p	0	0	1	1	0	0	2	3	0	0	**7**
miR-132-3p	0	0	0	0	0	0	1	1	0	1	**3**
miR-185-5p	0	0	3	0	0	1	3	6	0	0	**13**
miR-21a-5p	0	0	2	0	0	0	3	2	0	0	**7**
miR-381-3p	0	0	0	0	0	0	5	0	0	0	**5**
miR-92a-3p	0	0	0	0	0	0	1	1	0	0	**2**
miR-212-3p	0	0	2	0	0	0	1	3	0	1	**5**
miR-18a-5p	0	0	0	0	0	1	0	1	0	0	**2**
miR-363-3p	0	0	0	1	0	0	0	2	0	0	**3**
miR-665-3p	0	0	0	0	0	0	2	2	0	0	**4**
miR-7a-5p	0	0	0	0	0	0	1	0	0	0	**1**

Supplementary Table S3: Occurrences of keywords in article titles based on a Google Scholar search for each 13 miRNAs selected on the miRNA level and by target set testing. Each row corresponds to a specific miRNA, and the columns represent search terms used to query titles of publications. The numbers indicate how many times each search term appeared in the titles of publications associated with the respective miRNA.

microRNA ID	Database	Target Scan	Target Scan	Target Scan	Target Scan	mirdb	mirdb	mirdb	mirdb
	**Statistical Test**	**Fisher**	**Fisher**	**ROMER**	**ROMER**	**Fisher**	**Fisher**	**ROMER**	**ROMER**
	**Threshold**	**95**	**80**	**95**	**80**	**95**	**80**	**95**	**80**
miR-17-5p		X	X			X	X	X	X
let-7a-5p		X	X		X				
miR-15b-5p		X	X	X	X	X	X	X	X
miR-132-3p		X	X					X	X
miR-185-5p		X	X		X	X			
miR-21a-5p		X							
miR-381-3p		X	X						
miR-92a-3p		X	X	X	X	X	X		
miR-212-3p		X	X					X	X
miR-18a-5p		X		X	X	X	X		
miR-363-3p		X	X	X	X	X	X	X	X
miR-665-3p		X	X	X		X		X	
miR-7a-5p		X	X	X	X	X	X	X	

Supplementary Table S4: Robustness analysis of miRNAs selected by target set testing. The main analysis by target set testing was performed using target set information from the TargetScan database, Fisher's test and an prediction threshold of 95%, yielding 13 miRNAs that were also selected on the level of miRNA expression. The "X" symbol marks miRNAs that were also selected by other parameter settings of the target set testing pipeline.
